# Dairy Sanitation

**Published:** 1899-08

**Authors:** C. M. Day

**Affiliations:** Lincoln, Nebraska


### DAIRY SANITATION.

By C. M. Day, D.V.M.,
LINCOLN, NEBRASKA.

There is no subject of greater interest to the veterinary profession at the present time than sanitation, and no part of our national resources more in need of proper sanitation than the dairy. It is not the intention of the writer to discuss in detail and exhaustively this great and important subject, but to draw your attention briefly to the subject of sanitation relatively to the veterinarian, and especially the dairy.

It is a fact the price of horses has so diminished in the past few years as to render the practice of the veterinarian unprofitable and unremunerative. This condition of affairs is more readily seen in locations where the veterinarian has been depending upon the horse almost exclusively for his practice. This condition of affairs is considerably relieved where the practitioner has been able, either by his education or environments, to diversify his practice and assume all the various cases which would naturally be presented to a man who is able to treat the various kinds of animals and their diseases. But the application of sanitary law and police has done more, probably, to diversify the work of the veterinarian, and occupy his time and attention, than any other one subject in veterinary medicine.

The fact that so many of our profession in recent years have been placed on boards of health of various States and municipalities is quite sufficient to show the appreciation of the people and the ability and usefulness of the members of our profession as sanitarians. Well, it is so; for naturally, from the character of our education and training, there is no other body of professional men so thoroughly equipped and fitted to understand and see those grave dangers which beset the human race on many sides by the close relation between the diseases of animals and man.

The Bureau of Animal Industry, in the Department of Agriculture, in its meat-inspection division can best be likened, by the writer, to a great and powerful sanitation board, with its many veterinary inspectors, exerting its good and powerful influence toward guarding the source of our meat-supply, not only of this nation, but a large part of foreign trade, and thus lessening the danger and averting entirely many cases of disease and sickness among the people, by preventing, by condemnation, tons of diseased and dangerous meat from being placed on the market and sold to the unsuspecting public. In view of the great tendency of the people to turn to sanitation as a necessity in preventing the occurrence and ravages of many terrible contagious and infectious diseases, several veterinary medical colleges have provided means, and are paying considerable attention to the study of sanitation in their course.

It is found that animals on the farm kept under good sanitary conditions have greater power of resistance to the contagious and infectious diseases, and the ravages of the disease is somewhat mitigated. There is no doubt in the mind of the writer that if the porcine tribe were kept under thorough sanitary conditions they would have the power of resisting, to a certain degree, at least, the ravages of those terribly destructive diseases, cholera and swine-plague, and possibly in some cases prevent their appearance on the premises.

But I am afraid I am digressing too much from the original thought I wish to convey in this paper, that of sanitation in the dairy. It is this subject of sanitation in the dairy that has absorbed the thought and attention of our professional men and laymen as well. The necessity of proper sanitation in the dairy herd is apparent when we think of the millions of bacteria infesting the products of the dairy; and in milk they find a most natural and nutrient media for growth and development. To be sure there are a great number of bacteria naturally belonging to milk, bacteria necessary for the proper use and development of this lactic fluid into butter and cheese for the use of the people. To prevent the injurious bacteria from overcoming, misplacing and destroying the useful and beneficial action of the proper bacteria is the one great object in sanitation of the dairy.

In *Farmers' Bulletin*, No. 29, page 9, issued by the U. S. Department of Agriculture, it is stated that "considerably over two hundred distinct types of ordinary milk-bacteria have been described in literature up to the present time."

Of course, when milk is obtained and kept with proper care many of these bacteria develop, and do a good service to the butter and cheese-maker, by ripening the cream and giving a good flavor to both butter and cheese. But when we consider how liable the deleterious and even pathogenic germs are to get into the milk and overcome, destroy and prevent the beneficial action of the useful germs, it is plain to see and understand the necessity of proper and efficient sanitation of the dairy.

In regard to the pathogenic germs which are most likely to get into milk, develop and grow there rapidly, and when either the milk or its products are used by the people cause infection frequently to take place, and disease and death result—the germs causing diphtheria, typhoid fever, and tuberculosis are the most to be dreaded. The germ causing diphtheria (Klebs-Löffler bacillus) always finds its way into the milk by external contamination, and never through the internal agency of the cow. Its presence in milk has been traced to various circumstances. Among the many ways the germ gets into milk is the presence of the disease in some member of the dairyman's family, and absence of proper sanitary precautions, through negligence or ignorance of the danger; after attending her sick child, the mother going to strain and set away the new milk, without properly washing her hands and changing her clothing while engaged in both nursing and housekeeping, by allowing the milk to stand about in the buckets after it has been drawn at the cow-stable or milk-house, where cats and chickens get into it, as both cats and fowls are subject to diphtheria, and are liable to drop some of the germs into the milk with the saliva. These cases are not overdrawn, surely, as the writer has frequently seen such filthy, nauseating, and unsanitary conditions prevailing in many rural homes.

There are several ways in which typhoid bacillus finds its way into milk—some member of the family having a case of the fever, and the nurse not using the necessary precautions, the germs get spread and frequently find their way to the milk-house. Probably the more prolific source of the contamination of milk with the typhoid germ is the same by which the infection is generally spread among the people—through a contaminated water-supply. The water used to wash the milk utensils contaminates the milk by contaminating the utensils first.

Another disease to which I have not referred, and one often found contaminating the milk, is the germ of scarlet fever, producing many cases of the disease among children using the milk supplied by the dairy. The germ might find its way into the milk where a case of scarlet fever has affected one of the family, who has recovered and is able to be about, and the scales of cuticle (which usually peel off after the fever, and are laden with the noxious germs) fall into the milk if the convalescent subject should go about the milk-house; or should the scales even fall into the milk-buckets or cans the contamination would take place just the same, and be the cause of the dissemination of the disease where the milk is used.

The last and most important germ I think, of which I shall speak, is the bacillus tuberculosis. The danger of contamination with this germ occurs in two ways: first, contamination through an external source. This usually occurs by some unfortunate sufferer distributing the germ by his imprudent expectorations in places where it will come in contact either with the milk or milk utensils; or the germs may become lodged in the clothing of the sick dairyman, and becoming loosened fall into the milk while the person is engaged in care and handling. Whatever way the bacillus enters the milk the danger of infecting the consumer is just as great.

But the contamination of the milk of dairy herds by the internal source, or through the animals being diseased, is probably the most common way of contamination. After the dairy inspector has gone over all the ground, inspecting the entire dairy, its attendants, utensils, surroundings, etc., and succeeds in applying perfect sanitation to all these things, it would be proper, and just as important, to turn his attention to the inspection of the herd itself. This is a very important and a necessary part of dairy inspection, to have the herd in a healthful condition to insure wholesome and non-infected milk, as it has been unmistakably demonstrated many times by scientific investigators that tubercle bacilli are excreted in the milk of the cow, in many cases, when the animal has the disease, causing the consumers to take into their systems the deadly germs of the terrible disease, tuberculosis. To inspect the herds for this dreadful disease it is absolutely necessary to test with tuberculin, a substance, the result of the discovery of that world-wide famous scientist, Dr. Koch. Tuberculin has proven so efficacious in the diagnosis of tuberculosis in the bovine tribe that it has been universally recognized as the only safe and reliable method of detecting tuberculosis in cows.

When we read the statistics of deaths among the people in the world, and learn that one death in every eight is due to tuberculosis, it is sufficient to convince any reasonable person that it is the duty of every veterinary surgeon to agitate the question and educate the owners of dairy herds, till they realize the danger in which they are surrounded. To be sure, we have no complete data, and cannot tell exactly how many of these deaths in the human race are due to the infection coming from our dairy products; however, sufficient evidence has been produced in the estimable work of such noted investigators as Dr. James Law and others to convince anybody that many cases of tuberculosis in the human race are caused directly from the consumption of tubercle bacilli in dairy products.

It is not consideration alone for the vast destruction of the human race with this deadly germ, but there are pecuniary interests to be considered when a dairy herd has become infected with tuberculosis; in many instances it has proven disastrous to the owners; so many cows have succumbed to the disease that it has made the herd unprofitable.

As previously stated, the writer believes it the duty of our profession to bring this subject strongly and comprehensively before the people, and, in the light of our present knowledge on the subject, I believe they will be led to see and realize somewhat the grave dangers to our dairy industry which lies before them in this widespread, dreadful and persistent disease.

